# New Insights into the Fractional Order Diffusion Equation Using Entropy and Kurtosis

**DOI:** 10.3390/e16115838

**Published:** 2014-11-06

**Authors:** Carson Ingo, Richard L. Magin, Todd B. Parrish

**Affiliations:** 1 C.J. Gorter Center for High Field MRI, Department of Radiology, Leiden University Medical Center, Albinusdreef 2, 2333ZA Leiden, The Netherlands; 2 Department of Bioengineering, University of Illinois at Chicago, 851 S. Morgan St, Chicago, 60607, IL, USA; 3 Department of Radiology, Northwestern University, 737 Michigan Ave 16th Floor, Chicago, 60611 IL, USA

**Keywords:** entropy, kurtosis, fractional derivative, continuous time random walk, anomalous diffusion, magnetic resonance imaging, Mittag–Leffler function

## Abstract

Fractional order derivative operators offer a concise description to model multi-scale, heterogeneous and non-local systems. Specifically, in magnetic resonance imaging, there has been recent work to apply fractional order derivatives to model the non-Gaussian diffusion signal, which is ubiquitous in the movement of water protons within biological tissue. To provide a new perspective for establishing the utility of fractional order models, we apply entropy for the case of anomalous diffusion governed by a fractional order diffusion equation generalized in space and in time. This fractional order representation, in the form of the Mittag–Leffler function, gives an entropy minimum for the integer case of Gaussian diffusion and greater values of spectral entropy for non-integer values of the space and time derivatives. Furthermore, we consider kurtosis, defined as the normalized fourth moment, as another probabilistic description of the fractional time derivative. Finally, we demonstrate the implementation of anomalous diffusion, entropy and kurtosis measurements in diffusion weighted magnetic resonance imaging in the brain of a chronic ischemic stroke patient.

## 1. Introduction

The fundamental concept in continuous time random walk (CTRW) theory is to extend the diffusion equation, such that the fractional order partial derivatives can be used as the governing mathematical operators to describe the diffusion propagator, *P* (*x, t*):

(1) $$\frac{\partial^{\alpha }P(x,t)}{\partial t^{\alpha }}=D_{\alpha ,\beta }\frac{\partial^{\beta }P(x,t)}{\partial {|x|}^{\beta }},$$

that is, where ∂*^α^/*∂*t^α^* represents the Caputo fractional derivative in time for 0 *< α* ≤ 1, ∂*^β^/*∂*|x|^β^* represents the order of the Riesz fractional derivative in space for 1 *< β* ≤ 2 and *D_α,β_* is the generalized diffusion constant (distance*^β^*/time*^α^*) [1–3].

The justification for making use of the fractional derivative operators is to provide a mathematical means to interpolate from homogeneous and relatively simple systems that exhibit local, Gaussian behavior to heterogeneous and relatively complex systems that exhibit non-local, power-law behavior [2–8]. In the CTRW context, the order of the fractional operators, *α* and *β*, provides a description of a random walker’s likelihood to have broader distributions of waiting times and jump lengths, respectively, in comparison to classical Brownian motion. Because anomalous diffusion behavior implies a different probability model, one which postulates that the random walker sometimes halts between steps and sometimes takes larger or smaller steps, the likelihood, or weighting, placed on jumps and waiting times, which asymptotically follow simple inverse power laws: *t*^−(1+^*^α^*^)^ and *|x|*^−(1+^*^β^*^)^ [5]. As a result, bulk non-Gaussian motions emerge, and the mean squared displacement (MSD) no longer increases with a linear dependence on time. In the most basic form, the one-dimensional MSD is expressed by a composite power law as:

(2) $$\langle x^{2}(t)\rangle \sim t^{2\alpha /\beta }.$$

**Figure 1:**
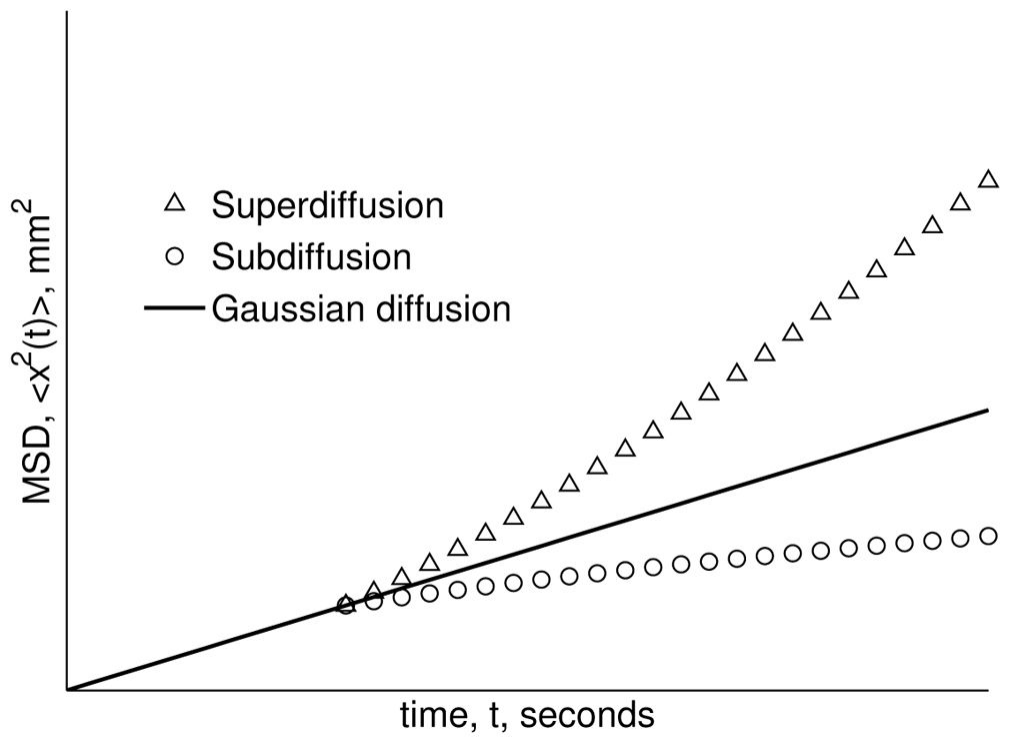
Sketches of the mean squared displacement for the cases of Gaussian diffusion (2*α/β* = 1), subdiffusion (2*α/β <* 1) and superdiffusion (2*α/β >* 1).

When *α* = 1 and *β* = 2, Equation (2) grows linearly with the diffusion time, and Equation (1) collapses to the integer-order partial differential equation to describe Gaussian diffusion. When the ratio 2*α/β <* 1, the MSD dynamics are, overall, subdiffusive, and when 2*α/β >* 1, the dynamics diverge to be described to have superdiffusive behavior (e.g., pseudo-transport) [3,5,7]. The MSD trajectories of Gaussian, sub- and super-diffusion are illustrated in Figure 1, where in the case of subdiffusion, the variance grows slower than the Gaussian case, where in the case of superdiffusion, the variance increases faster than the linear growth of the Gaussian. In the context of the MSD, when the power law properties of the random walk distributions are connected with the order of the fractional derivatives, *α* and *β* encode either a fading memory in time or extended neighbor interactions in space as a consequence of the mathematical operators in the fractional partial differential equation. As a new perspective to view the utility of fractional order calculus, we consider power law diffusion dynamics from the perspective of information theory and make use of entropy as a measurement tool for the characteristic function (CF) of the diffusion propagator. Since the fractional derivative is a heuristic tool that includes, in its very definition, a distribution of time and space constants in order to represent multi-scale systems, we find that fractional order models convey more information, via entropy, about the underlying structure and dynamics of complex systems. Furthermore, we compute the kurtosis of the time-fractional solution to the diffusion equation as an alternative measure to demonstrate how the fractional operator encodes heterogeneous, non-Gaussian dynamics. Finally, we demonstrate the utility of the fractional order derivative, entropy and kurtosis to provide new contrast to characterize biological tissue microstructure beyond the classical model for diffusion MRI measurements in the brain of a chronic ischemic stroke patient.

## 2. Experimental Section

### 2.1. Theory

**Figure 2:**
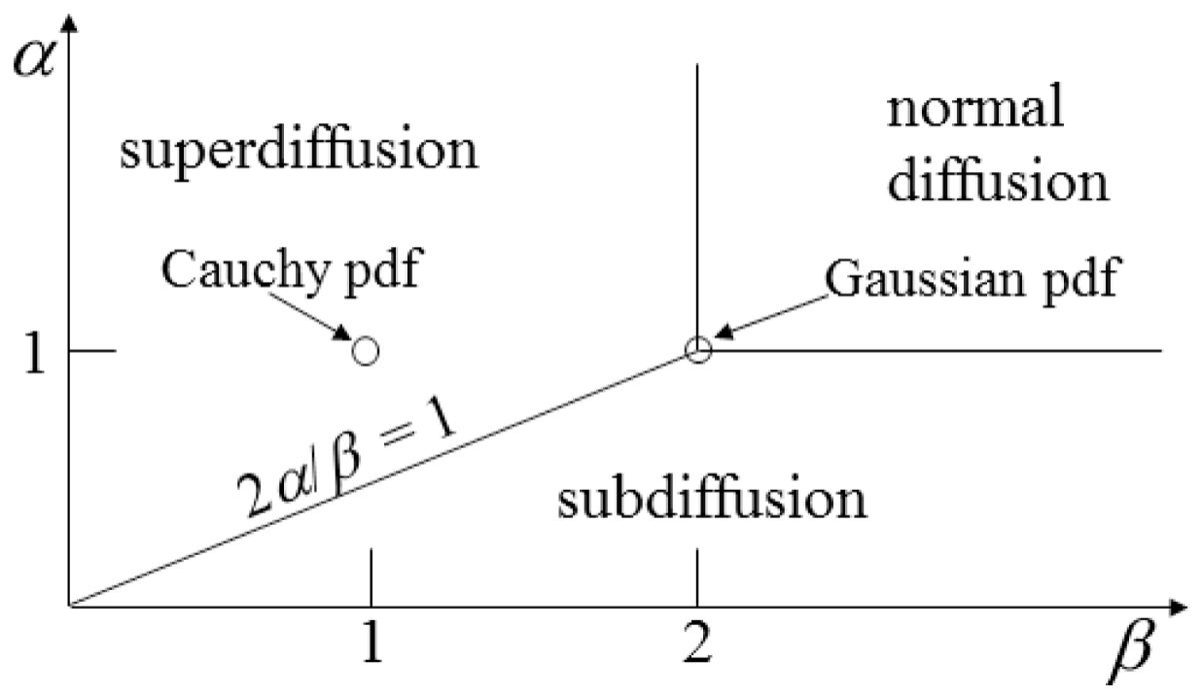
Anomalous diffusion phase diagram with respect to the order of the fractional derivative in space, *β*, and the order of the fractional derivative in time, *α*.

In the general case, the order of the fractional partial derivatives, *α* and *β*, can span a range of values that encompass processes characterized as both subdiffusion and as superdiffusion in the diffusion phase diagram shown in Figure 2. This distinction is made by comparison with Brownian motion and the second moment of the Gaussian distribution, which increases as a linear function of time (*α* = 1 and *β* = 2). This diagram is formally derived in CTRW theory, where *P* (*x, t*) expresses the probability density of finding the molecule at position *x* at time *t*, assuming that at *t* = 0, all of the material is concentrated at the origin, where *P* (*x,* 0) = *δ*(*x*) as a Dirac delta function[1,5].

Conveniently, utilizing Fourier and Laplace transforms, Equation (1) can be succinctly expressed in the form, *P* (*x, t*) → *p*(*k, s*):

(3) $$p(k,s)=\frac{1}{s+D_{\alpha ,\beta }s^{1-\alpha }{|k|}^{\beta }}.$$

Then, by applying the inverse Laplace transformation to Equation (3), we obtain a simple expression for the relaxation for each wavenumber, k:

(4) $$p(k,t)=E_{\alpha }\left(-D_{\alpha ,\beta }{|k|}^{\beta }t^{\alpha }\right).$$

The symbol *E_α_* represents the single-parameter Mittag–Leffler function (MLF), which describes a generalized power law decay for the range, 0 *< α <* 1, and a damped, but oscillating function for 1 *< α <* 2 [9,10]. Specific functions encapsulated by the MLF are a monoexponential decay when *α* = 1 and the cosine function when *α* = 2. Thus, for a fixed wavenumber and diffusion coefficient, Equation (4) corresponds to the time decay (with or without oscillations) of one spectral component. As the order of the fractional time derivative *α* decreases from one, there is a shift away from the single exponential decay toward an apparent multi-exponential relaxation in time. In the case of *α* = 1 and *β* = 2, we have the characteristic form for a Gaussian distribution,

(5) $$p(k,t)=\exp(-D_{1,2}{|k|}^{2}t).$$

Clearly, as either the time or the diffusion coefficient increase, the spread of wavenumbers decreases, which, through the scaling property of the Fourier transform, gives a correspondingly wider distribution in space. Conversely, as the time or the diffusion coefficient decrease, the spread of the wavenumbers increases and the distribution of the diffusion process in space is more localized. This phenomena is described in the space-time domain for normal diffusion by the probability diffusion function (pdf) of the Gaussian:

(6) $$P(x,t)=\frac{1}{\sqrt{4\pi D_{1,2}t}}\exp\left(-\frac{x^{2}}{4D_{1,2}t}\right),$$

where the mean squared displacement in space is simply the second moment (or variance),

(7) $$\langle x^{2}(t)\rangle =2D_{1,2}t.$$

In comparison to the Gaussian case, there is the condition of time-fractional subdiffusion, 0 *< α <* 1 and *β* = 2. Equation (3) becomes,

(8) $$p(k,s)=\frac{1}{s+D_{\alpha ,2}s^{1-\alpha }{|k|}^{2}},$$

and Equation (4) becomes,

(9) $$p(k,t)=E_{\alpha }\left(-D_{\alpha ,2}{|k|}^{2}t^{\alpha }\right).$$

By first utilizing the simple form of the Laplace–Fourier solution to time-fractional subdiffusion in Equation (8), the MSD can be computed by taking the second derivative of Equation (8) with respect to *k* as the limit of *k* → 0 and then performing a Laplace inversion,

(10) $$\langle x^{2}(t)\rangle =L^{-1}\lim_{k\to 0}\{-\frac{d^{2}p(k,s)}{dk^{2}}\},$$

which gives,

(11) $$\langle x^{2}(t)\rangle =L^{-1}\{2D_{\alpha ,2}s^{-(\alpha +1)}\},$$

and utilizing the common Laplace and time domain transform pair,

(12) $$\frac{t^{\alpha }}{\Gamma (\alpha +1)}=L^{-1}\{s^{-(\alpha +1)}\},$$

yields the form of the MSD,

(13) $$\langle x^{2}(t)\rangle =\frac{2D_{\alpha ,2}}{\Gamma (\alpha +1)}t^{\alpha },$$

as reported in [5].

For the antithetical case of superdiffusion, *α* = 1 and *β <* 2, Equation (3) becomes,

(14) $$p(k,s)=\frac{1}{s+D_{1,\beta }{|k|}^{\beta }},$$

and applying the inverse Laplace transform yields a stretched exponential about *k*,

(15) $$p(k,t)=\exp\left[-D_{1,\beta }{|k|}^{\beta }t\right],$$

which is the CF for a centered, symmetric Levy distribution [8]. Unfortunately, Equation (15) cannot be analytically expanded at *k* = 0 when *β <* 2, and so, the second moment is undefined for the diffusion propagator, *P* (*x, t*). In contrast, the CF for subdiffusion in Equation (8) has a closed form representation of the MSD in Equation (13), so it is possible to compute the kurtosis for the diffusion propagator as a function of the order of the fractional time derivative as follows.

### 2.2. Kurtosis

Qualitatively, kurtosis is a measure to nonspecifically describe the peakedness and/or heavy tail shape in a probability distribution via the standardized fourth moment [11]. For example, a Gaussian probability distribution function (pdf) has a kurtosis value of three, whereas the hyperbolic secant pdf has a kurtosis value of five, as its shape is both more peaked and heavier-tailed than the Gaussian shape. A convenient index called excess kurtosis is defined as the difference between the estimated kurtosis value for a given distribution with the value of the Gaussian pdf (*i.e.*, three), such that the excess kurtosis of a Gaussian pdf is zero. In the context of the mathematical definition of excess kurtosis, diffusion processes with positive excess kurtosis imply that the diffusion propagator has a standardized fourth moment, which is broader than the Gaussian case,

(16) $$K\equiv \frac{\langle x^{4}\rangle }{{\langle x^{2}\rangle }^{2}}-3.$$

Returning back to the formalism of operating in the Laplace domain, the fourth moment of Equation (8) can be expressed as,

(17) $$<x^{4}(t)>=L^{-1}\{24{\left(D_{\alpha ,2}\right)}^{2}s^{-(2\alpha +1)}\},$$

and inverting into the time domain using the transform in Equation (12) gives,

(18) $$<x^{4}(t)>=\frac{24{\left(D_{\alpha ,2}\right)}^{2}}{\Gamma (2\alpha +1)}t^{2\alpha }.$$

Inserting Equation (13) and Equation (18) into Equation (16), the excess kurtosis of the MLF for time-fractional subdiffusion is,

(19) $$K_{\mathrm{MLF}}=6\frac{\Gamma^{2}(\alpha +1)}{\Gamma (2\alpha +1)}-3.$$

**Figure 3:**
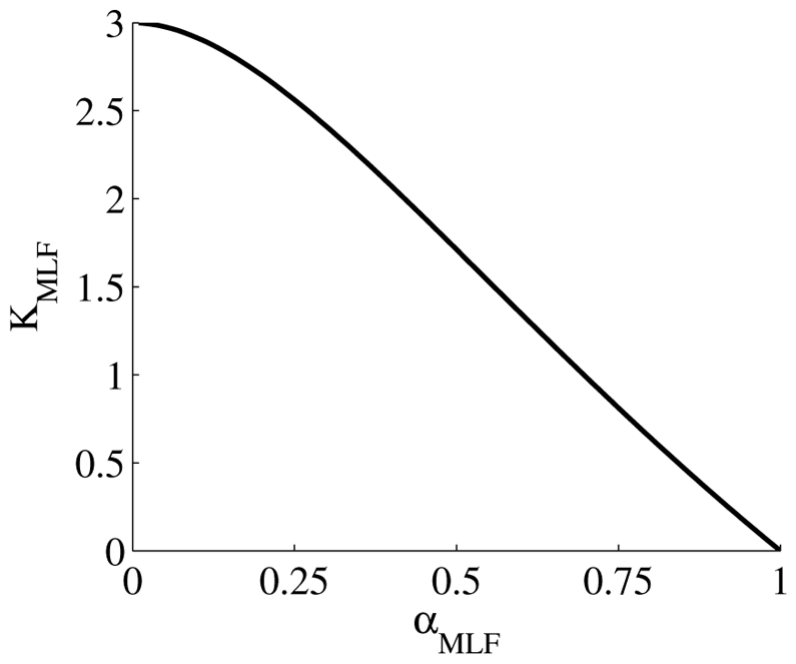
Plot of Equation (19) for the kurtosis, *K_MLF_*, computed in the Mittag–Leffler representation of subdiffusion *versus* the time-fractional derivative, *α*.

When *α* = 1, the MLF becomes the monoexponential CF and *K_MLF_* = 0. For 0 *< α <* 1, *K_MLF_ >* 0 with the maximum excess kurtosis value limited to max(*K_MLF_*) = 3 when *α* → 0. Equation (19) is plotted in Figure 3, which shows a nearly inverse linear relationship between *K_MLF_* and *α*.

As mentioned above, the CF for Equation (15) cannot be expanded as a Maclaurin series (*k* = 0). However, as Equation (15) exists in closed form as a stretched exponential function, it is possible to consider entropy in the Fourier domain. Furthermore, in general, since *p*(*k, t*) is available in closed form as the MLF in Equation (4) for all diffusion regimes, we are able to compute the entropy for the cases of Gaussian, sub- and super-diffusion from the perspective of information theory.

### 2.3. Entropy

The most fundamental formula to compute entropy, or the amount of uncertainty in a discrete pdf, *P* (*x*) can be measured with,

(20) $$H_{x}\equiv -\sum_{i=1}^{N}P(x_{i})ln(P(x_{i})),$$

where *P*(*x_i_*) are the individual samples in the the discrete pdf and *H_x_* is the Shannon information entropy [12]. With the consideration of information formulated in the context of statistical uncertainty, we have a tool to compare systems governed by differing stochastic processes. For example, when comparing two distributions, both normalized with the same full-width, half maximum values, the heavier-tailed distribution can be shown to have greater information entropy, pertaining to greater statistical uncertainty. Another approach to measure the uncertainty in a system is to analyze the characteristic function in terms of the Fourier transform in space, *P* (*x*) → *p*(*k*), with spectral entropy,

(21) $$H_{k}\equiv -\sum_{i=1}^{N}\frac{\hat{p}(k_{i})ln(\hat{p}(k_{i}))}{ln(N)},$$

where *p̂*(*k_i_*) = *p*(*k_i_*)p^*^(*k_i_*) reflects the individual wavenumber’s contribution to a normalized power spectrum of the Fourier transform, *p_k_*, and the term, *ln*(*N*) (*i.e.*, discrete uniform distribution of *N* samples), is a normalization factor applied so that the spectral entropy, *H_k_*, is between zero and one [13,14]. Furthermore, as Equation (21) is generally defined to measure the uncertainty of a characteristic function, we can adapt this formalism to compare the CFs for anomalous diffusion,

(22) $$H[\hat{p}(k,t)]\equiv -\sum_{i=1}^{N}\frac{\hat{p}{\left(k,t\right)}_{i}ln(\hat{p}{\left(k,t\right)}_{i}}{ln(N)}.$$

By inserting the characteristic function in Equation (4) into Equation (22), the entropy in arbitrary diffusion processes can be measured. Furthermore, in the context of MRI, in which the reconstructed signal is considered as the Fourier transform in space, Equation (22) can be rewritten as a model for the diffusion MRI weighting factor *b* ≡ *q*^2^Δ̄, where by convention, *q* is diffusion gradient strength sensitization (e.g., units of mm^−1^) and Δ̄ is the effective diffusion time (e.g., units of ms) to be rewritten as,

(23) $$H[\hat{p}(q,\bar{\Delta })]\equiv -\sum_{i=1}^{N}\frac{\hat{p}{\left(q,\bar{\Delta }\right)}_{i}ln(\hat{p}{\left(q,\bar{\Delta }\right)}_{i}}{ln(N)}.$$

to demonstrate the utility of Equations (9), (19) and (23) as models to characterize biological tissue in non-Gaussian diffusion MRI measurements. Furthermore, we examine, via spectral entropy (Equation (22)) the properties of the MLF to encapsulate the special cases of time- and space-fractional diffusion processes, which, in general, are shown to have more information content than the case of a Gaussian diffusion process.

### 2.4. Methods for Diffusion MRI Experiments

**Figure 4:**
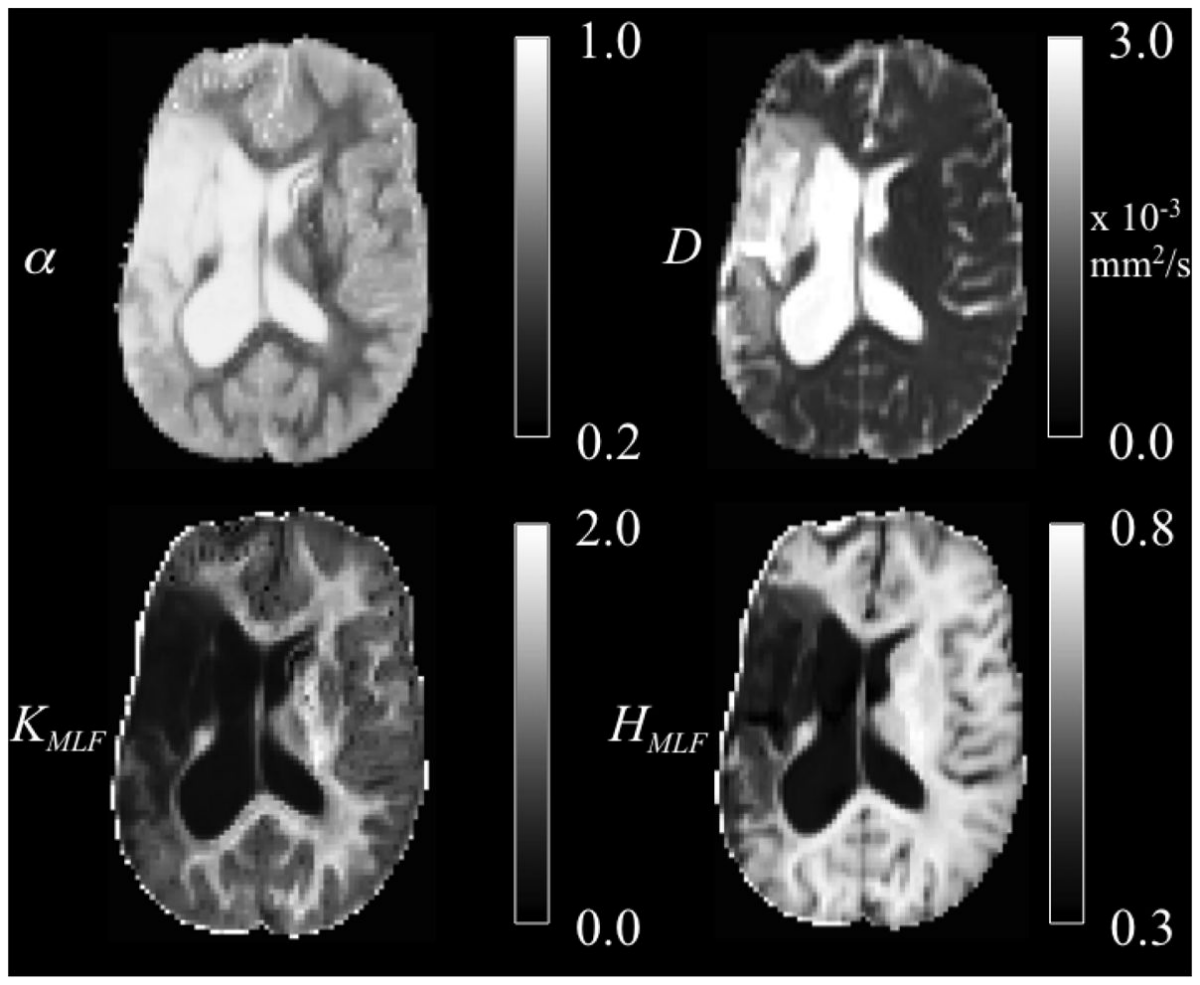
Trace parameter maps of *α*, *D*, *K_MLF_* and *H_MLF_* for an axial slice through a brain of a chronic stroke patient.

To demonstrate the utility of the fractional order of the time derivative, entropy and kurtosis in the diffusion of water within biological tissue, one patient with chronic ischemic stroke was scanned on a 3 Tesla Siemens Trio MRI scanner (Siemens Medical Solutions, Erlangen, Germany). Diffusion-weighted spin echo-echo planar imaging (SE-EPI) experiments were performed with the following pulse sequence parameters: echo time *T E* = 102 ms, repetition time *T R* = 6 s, diffusion time of Δ = 41.2 ms, pulse duration of *δ* = 40.6 ms Δ̄ = Δ − *δ/*3 = 27.7 ms, diffusion weightings of *b* = 0, 500, 1000, 3000, 4000 s/mm^2^, 3 orthogonal diffusion weighted directions, number of averages *NA* = 6, in-plane voxel resolution = 2 *×* 2 mm, voxel thickness = 4 mm, 20 axial slices, scan time *~* 6 minutes. The raw diffusion weighted data were Rician noise corrected by estimating the variance (*σ*^2^) in the signal intensity of the ventricle at each *b*-value, such that $S_{rn}=\sqrt{S^{2}-\sigma^{2}}$. The Rician noise-corrected diffusion weighted images were skull-stripped utilizing the Brain Extraction Tool [15]. All skull-stripped and Rician noise-corrected diffusion weighted images were co-registered to the *b* = 0 image space using statistical parametric mapping software (SPM8). Using the Levenberg–Marquardt minimization algorithm in MATLAB (Natick, MA, USA), the average of the three diffusion weighted direction data were fit on a voxel-wise basis to Equation (9) with the MLF algorithm in [16,17]. Following estimations of *D* and *α*, the excess kurtosis, *K_MLF_*, was computed using the conversion provided in Equation (19). Following estimations of *D* and *α*, the CF in Equation (9) for *p*(*k, t*) was constructed using *N* = 100 increments arrayed over variable *b*-values between 0 and 10 000 s/mm^2^. Then, the entropy (defined in Equation (22)) in the diffusion process, as modeled by the MLF, was computed as *H_MLF_*. The isotropic parameter maps of *D*, *α*, *K_MLF_* and *H_MLF_* for the same axial slice through the stroke patient’s brain are shown in Figure 4.

### 2.5. Methods to Evaluate Entropy in the Mittag–Leffler Function

**Figure 5:**
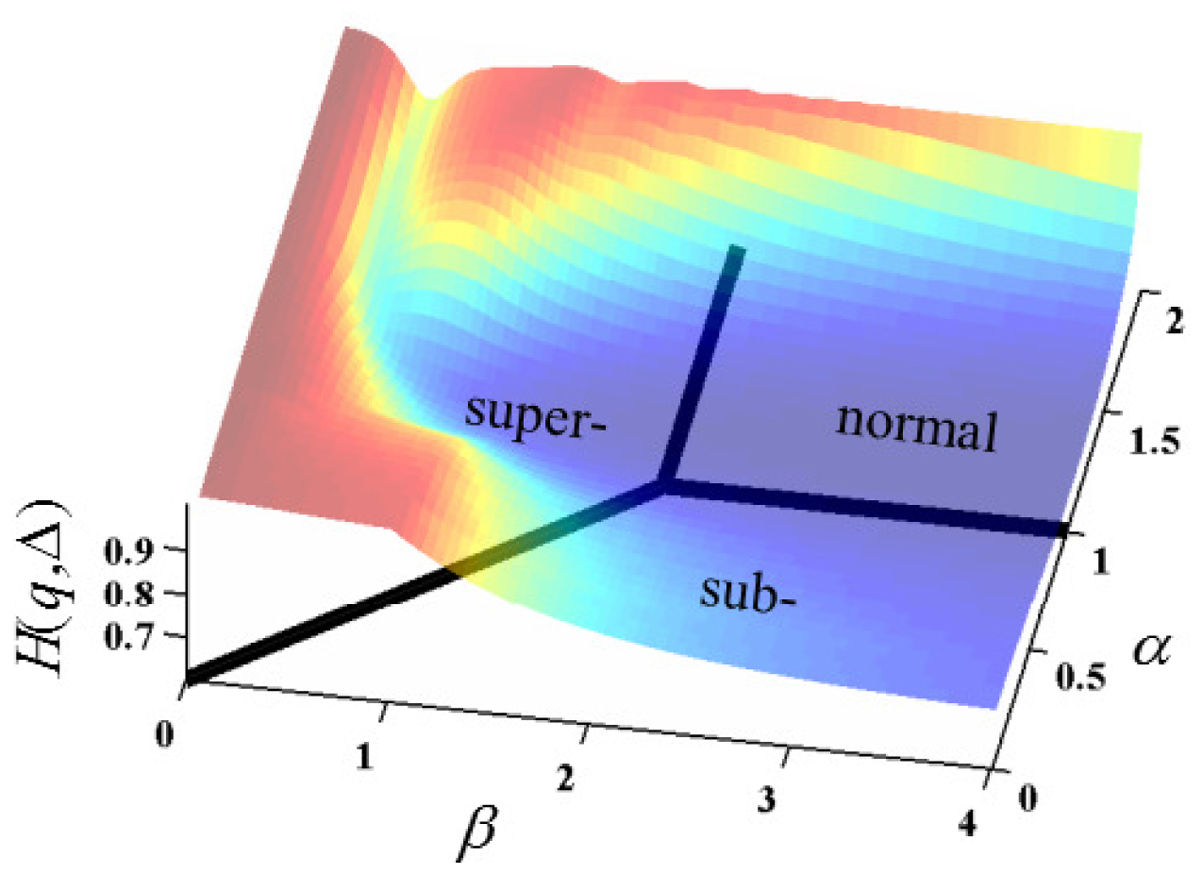
Spectral entropy surface plot for the Mittag–Leffler function (MLF) in Equation (4) with respect to the order of the fractional space derivative, *β*, and the order of the fractional time derivative, *α* (*D_α,β_* = 1*, t* = 1). The floor of the plot corresponds to the anomalous diffusion phase diagram.

**Figure 6:**
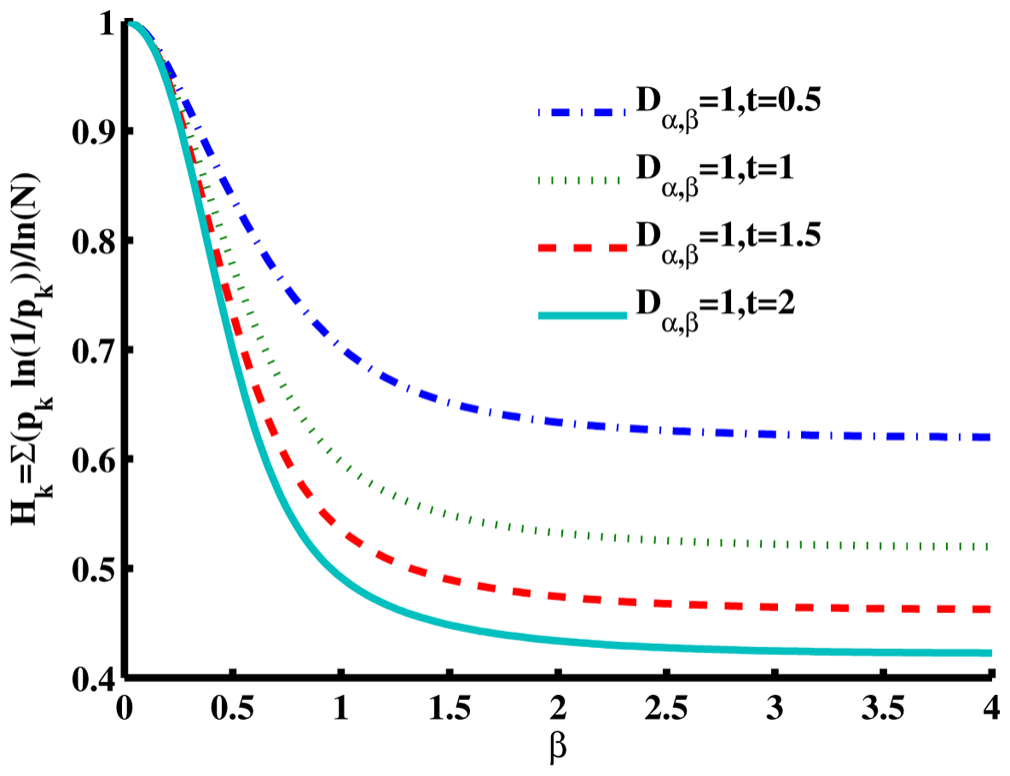
Spectral entropy for Equation (15) with respect to the order of the fractional space derivative, *β*, with diffusion time cases where *t* = 0.5*,* 1*,* 1.5*,* 2 for *α* = 1 and *D*_1_*_,β_* = 1.

**Figure 7:**
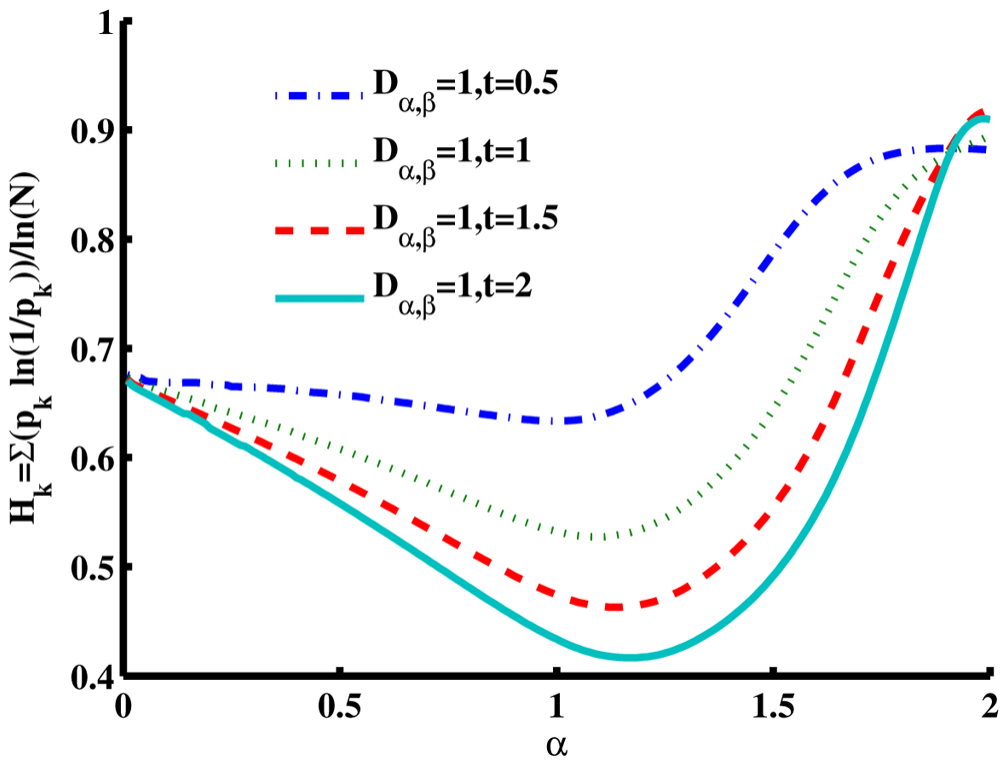
Spectral entropy for Equation (9) with respect to the order of the fractional time derivative, *α*, for four diffusion time cases where *t* = 0.5*,* 1*,* 1.5*,* 2 for *β* = 2 and *D_α,_*_2_ = 1.

**Figure 8:**
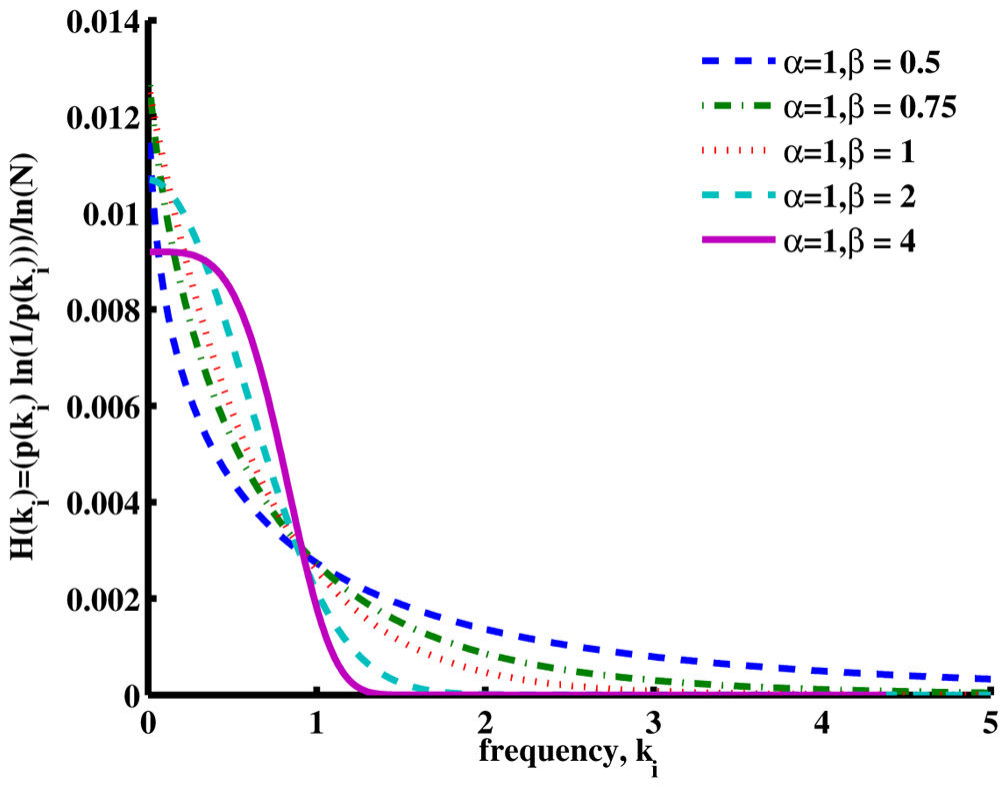
Plot of the individual wavenumber contributions to the spectral entropy of Equation (15) when the order of the fractional space derivative *β* = 0.5*,* 0.75*,* 1*,* 2*,* 4 for *α* = 1, *D*_1_*_,β_* = 1 and *t* = 1.

**Figure 9:**
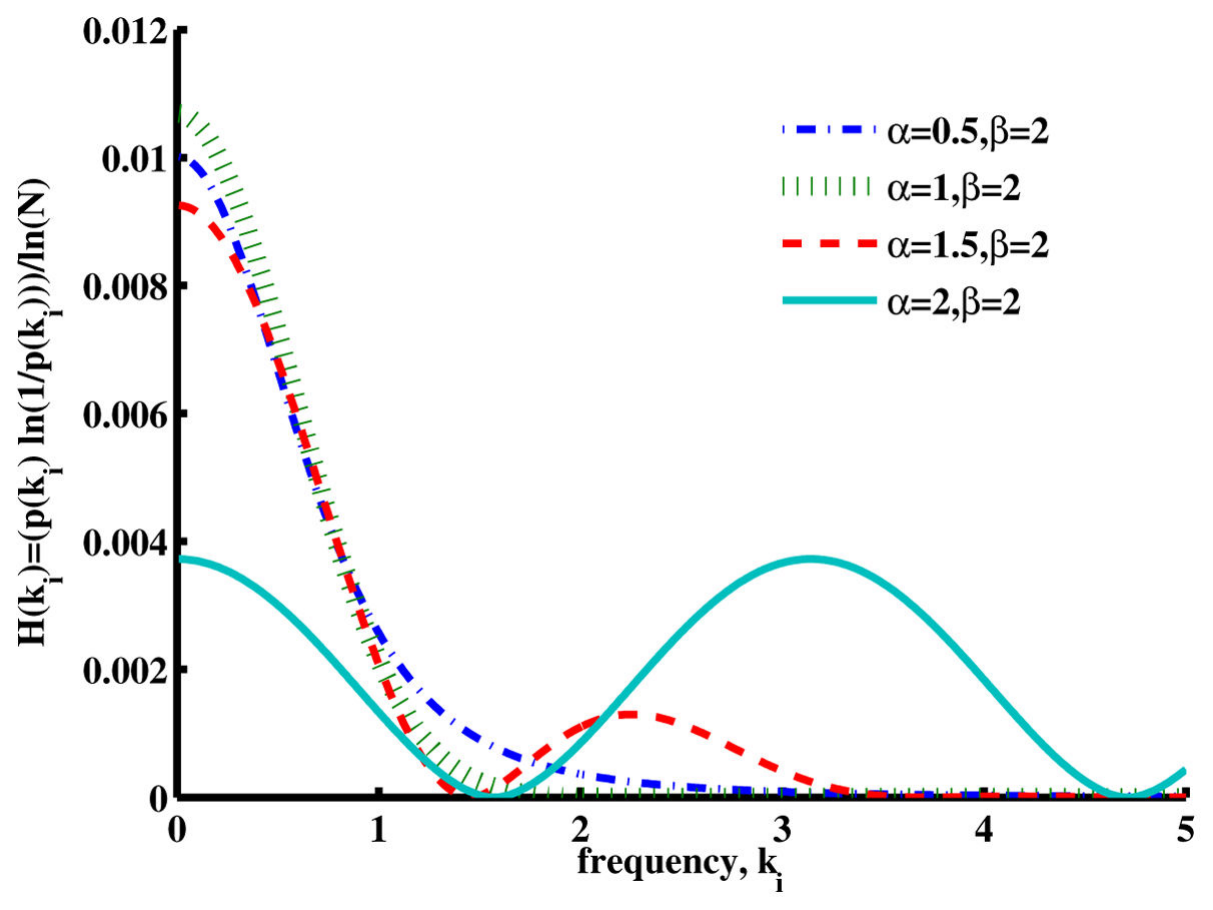
Plot of the individual wavenumber contributions to the spectral entropy of Equation (9) when the order of the fractional time derivative *α* = 0.5*,* 1*,* 1.5*,* 2 for *β* = 2, *D_α,_*_2_ = 1 and *t* = 1.

We computed the entropy for the CTRW model of diffusion by insertion of Equation (4) into Equation (21) by considering cases of 0 *< α* ≤ 2 and 0 *< β* ≤ 4. Using MATLAB, the MLF was computed using the algorithm in [16,17]. For simplicity, the units are arbitrary with a fixed diffusion coefficient of *D* = 1; the diffusion times are evaluated at four cases *t* = 0.5 – 2, and the wavenumbers are incremented in an array *k_i_* = 0 *–* 5 for *N* = 500 points. The overall results for entropy in the MLF are presented in Figure 5 as a three-dimensional entropy surface drawn above a plane defined by the positive values of *α* and *β*. The floor of the plot is essentially the phase diagram shown in Figure 2. Figures 6 and 7 plot the entropy in the diffusion process as a function of the order of the fractional derivative in space and time, respectively. Figures 8 and 9 plot the individual contributions to the total entropy of the diffusion process as a function of the wavenumber, *k_i_*, for selected cases of *α* and *β*.

## 3. Results and Discussion

### 3.1. Diffusion MRI Experiments

The ischemic tissue (IT), in the right hemisphere of the patient’s brain (left side of the image), has a diffusion coefficient, *D*, value (*~* 3 *×* 10^−3^ mm^2^/s), which is similar to the typical value found for the cerebral spinal fluid (CSF) of the ventricles. As can be seen in the contralateral hemisphere, prior to the onset of the stroke, the brain slice would have appeared symmetrical with white matter (WM) and gray matter (GM) voxels. However, as these data were acquired *~* 2 years following onset, the IT microstructure has degenerated (necrosis), such that the bulk diffusion coefficient has increased to an unhindered value. Furthermore, the diffusion in the IT is close to Gaussian as *α ~* 1, indicating a monoexponential behavior, which is also the case for the CSF. The trace values for *D* in the healthy WM and GM are *~* 1*/*3 of the values in the IT and CSF, with the WM possessing an overall slower diffusion than measured in the GM.

As the scale in the *D* map in Figure 4 spans 3 *×* 10^−3^ mm^2^/s, the contrast between WM and GM is difficult to discern; however, in the *α* map, the WM/GM contrast is clearly visible with the WM demonstrating more subdiffusive behavior compared to the GM. The *K_MLF_* map also has clearly visible GM/WM contrast and appears as a negative image in the *α* map, due to the nearly inverse relationship between *K_MLF_* and *α* in Equation (19). The entropy, *H_MLF_*, map provides a stable image in which there is visible and smooth GM/WM contrast, with the IT and CSF exhibiting low values of entropy due to the unhindered Gaussian diffusion dynamics. Interestingly, voxels exhibiting low values of *α*, representing highly subdiffusive dynamics, also have relatively high entropy estimations (particularly in the WM) to indicate a diffusion propagator with a heavy-tailed pdf. In correspondence with entropy, there is high kurtosis estimated for the diffusion propagator pdf in regions of low values of *α*. However, kurtosis and entropy are not interchangeable measures of the diffusion propagator pdf, evident not only in the images shown in Figure 4, but also mathematically distinct in the forms of Equations (19) and (22). Specifically, in consideration of the CF, *α* and *K_MLF_* are measures of the deviation from a monoexponential form with a rate of the diffusion coefficient, *D*, whereas entropy considers the entire CF, which includes both the monoexponential component, *D*, and the non-Gaussian component, *α*, of the diffusion profile. As kurtosis is defined as the normalized fourth moment in Equation (16), it can be readily seen that the variance, or *D*, is canceled out when dividing Equation (18) by Equation (13).

Utilizing the moment expansion of the time-fractional form of the MLF in Equations (13) and (18) provides a direct link for kurtosis to subdiffusion through the Γ function and *α*. As Equation (9) is an analytic and monotonically decreasing function, the MLF provides the opportunity to more completely sample wavenumber and diffusion time distributions, to more accurately estimate the true kurtosis of the diffusion propagator, which is an advantage compared to other approaches that estimate kurtosis through Taylor expansion of the exponential function [18].

### 3.2. Measuring the Mittag–Leffler Function with Spectral Entropy

In Figure 5, the overall shape of the surface resembles plateaus of high entropy at non-integer values of *α* and *β* with a canyon of low entropy (near *α* = 1) that flows in the direction of increasing *β*. The Gaussian case (*α* = 1 and *β* = 2) is at a minimum for permutations of *α* ≤ 1 and *β* ≤ 2. Of course, the specifics of the cross-sectional shape and entropy values of the surface is subject to change, with values chosen for the values of *D* and *t* as detailed in the analyses provided by Figures 6–9.

Figure 6 is a slice of the spectral entropy surface (for *α* = 1 and four values of time) from the *β* = 0 rim out to the distance of *β* = 4. Selecting one case of the argument, say *D*_1_*_,β_* = 1*, t* = 1, and starting at *β* = 2, we observe that the entropy increases as *β* gets smaller, with an approximately 20% increase in the normalized spectral entropy when *β* = 1 (the Cauchy distribution), whereas travel in the direction of increasing *β* is mostly flat by this measure of entropy. From the Gaussian location, *β* = 2, the entropy appears to converge to a value near 0.5 for increasing *β*, while for decreasing *β*, the entropy increases in a monotonic manner at short times. The effect of increasing diffusion time (or larger values of the diffusion coefficient) results in a decrease in the magnitude for the normalized entropy values, as demonstrated going from *t* = 0.5 to *t* = 2. In the phase diagram (Figure 2: *α* = 1 and *β <* 2), Figure 6 evaluates the case of super-diffusion, and it is encouraging that this perspective portrays this regime, which includes the CF of the Cauchy distribution, as one of higher entropy (in comparison with the Gaussian diffusion case).

Figure 7 is a slice of the spectral entropy surface (for *β* = 2 and four values of time) from the *α* = 0 rim out to the distance of *α* = 2. Selecting one case of the argument, say *D_α,_*_2_ = 1*, t* = 1, and starting at *α* = 1, we observe the entropy increasing in both directions, overall. Again, the depth of the minimum grows for longer times, but in this cross-sectional view, the location is in the direction of higher values of *α*. As is shown in the phase diagram (Figure 2), when *β* = 2, values of *α >* 1 are in a region of super-diffusion and values of *α <* 1 are in a region of subdiffusion. Furthermore, in Figure 7, we observe that for a specific value of time (and diffusion coefficient constant), the entropy generally increases (from the Gaussian diffusion case of *α* = 1) as the value of *α* increases, as well as as it decreases. Thus, both higher and lower values of the order of the fractional derivative *α* (relative to *α* = 1) give higher entropy values.

In both Figure 6 and Figure 7, it is interesting to note that as the product of the diffusion coefficient and the time increases, the spectral entropy decreases. Mathematically, this behavior is consistent with the Fourier- transform duality between the space and the wavenumber (spatial frequency) domains, in which the diffusion coefficient and time, *Dt*, change position from the denominator to the numerator of the argument (see Equation (5) and Equation (6) for the case of a Gaussian pdf). Thus, as diffusion time increases, in the framework of the space domain, we expect the distribution to widen and the entropy to increase (increasing variance for the Gaussian). Conversely, as the diffusion time increases, in the framework of the spatial frequency domain, we expect the distribution to narrow and the entropy to decrease. From a CTRW physical model perspective, as the diffusion time increases in the spatial domain, we argue that the distribution widens and the entropy increases as a dynamic measure by which the uncertainty in predicting the location of the diffusing particle increases. As such, more information is required to specify the spatial location of the particle as the diffusion time increases. Conversely, as the diffusion time increases in the spatial frequency domain, we argue that the distribution narrows and the entropy decreases as a dynamic measure by which the amount of information to be gained about the diffusion environment decreases. Therefore, as the CTRW process progresses in time, the environment becomes completely explored, and no new information can be captured about the system, albeit at the cost of maximum uncertainty about the particle’s location in space.

In order to examine further the factors that are summed in Equation (22), we have plotted (for a fixed diffusion coefficient and time) a single spectral entropy term as a function of the wavenumber for a series of *β* values in Figure 8 (stretched exponential function) and a series of *α* values in Figure 9 (stretched MLF). Here, for *β* = 2, the characteristic Gaussian shape is apparent, and as *β* decreases into the domain of super-diffusion, the spectrum appears to narrow, but in fact, due to the long power law tail, it actually spreads out, expanding the number and the range of higher wavenumber components. The sum of many of these terms can be interpreted as adding information to the corresponding spatial distribution, increasing its variance and its entropy. Furthermore, in this figure, we note that the Cauchy distribution (*β* = 1) has, in comparison with the Gaussian distribution, a wider spectral distribution, with a corresponding increase in spatial complexity and entropy.

The spectral entropy plotted in Figure 9 has similar features. For *α* = 1, the expected Gaussian distribution of spectral entropy is apparent. When *α* is reduced to 0.5, the spectra expand (higher uncertainty, higher entropy), and when *α* is increase to 1.5 and to 2, an oscillation appears in the spectra due to the behavior of the MLF, which again pushes more wavenumber components into the higher range. Such components would be expected to add uncertainty and entropy to the spatial distribution. In the case of *α* = 2, we have a cosine function in the wavenumber, which corresponds to a single, very small spatial feature (a Dirac Delta function) in space.

## 4. Conclusions

In this study, we have shown that entropy can be used as a measure of the information content, or uncertainty, in the diffusion propagator pdf for cases of Gaussian, sub- and super-diffusion. Both the space (*β*) and the time (*α*) fractional order dependence are expressed separately as special cases governed by the Mittag–Leffler function. The classical, Gaussian case of normal diffusion (*α* = 1; *β* = 2) is at a minimum of a surface plot of the spectral entropy for the selected range of *α* and *β* in the Mittag–Leffler function. In all directions from the minimum on this surface, the entropy increases, both for increasing and for decreasing values of the orders of fractional differentiation. When either *α* or *β* diverge from the Gaussian case, (*α* = 1; *β* = 2), the spectra for each component of the total entropy expand or contract in a manner that captures greater overall information about the system. There is an overall reduction in the total spectral entropy as time (or the diffusion coefficient) increases, corresponding to a wider spatial distribution of the individual diffusing components, which is consistent with the noted contraction of the wavenumbers in the Fourier spectral domain. Additionally, for the case of time-fractional subdiffusion in which the second and fourth moments are finite, we have presented a new formulation for excess kurtosis, *K*, which has a nearly linear increase when the time derivative, *α*, decreases away from the integer order. Finally, we performed diffusion weighted MRI measurements in the brain of a patient with chronic ischemic stroke in order to demonstrate the utility of *α*, *K_MLF_* and entropy *H_MLF_* measures to provide additional information about biological tissue microstructure beyond the classical diffusion coefficient, *D*.
